# Is There any Correlation Between Pulmonary and Coronary Involvement in Diabetic Patients?

**DOI:** 10.5812/ijem-160600

**Published:** 2025-07-31

**Authors:** Mahmoud Parham, Mohammad Aghaali, Seyed Hassan Adeli, Ahmad Reza Bagheri, Masoud Sadeghi, Akram Asghari

**Affiliations:** 1 Clinical Research Development Unit, Shahid Beheshti Hospital, Qom University of Medical Sciences, Qom, Iran; 2 Department of Family and Community Medicine, School of Medicine, Qom University of Medical Sciences, Qom, Iran; 3 Department of Cardiology, School of Medicine, Qom University of Medical Sciences, Qom, Iran

**Keywords:** Diabetes Mellitus, Coronary Artery Disease (CAD), Macrovascular Complications, Spirometry, Age-Related Changes

## Abstract

**Background:**

Diabetes is a major global health issue, affecting over 8.5% of adults worldwide and significantly increasing the risk of cardiovascular diseases (CVDs). However, the association between diabetes-related macrovascular complications, such as coronary artery disease (CAD), and pulmonary function remains unclear.

**Objectives:**

The present study was designed to investigate the association between obstructive and restrictive lung patterns and the severity of coronary artery involvement.

**Methods:**

A cross-sectional study was conducted at Shahid Beheshti Hospital, Qom, Iran, between April 2019 and August 2021. A total of 211 diabetic patients who underwent coronary angiography (CAG) were selected. They were divided into two groups: Sixty-nine patients in group 1 (non-significant CAD) and 142 patients in group 2 (significant CAD). Spirometry was performed to assess forced expiratory volume (FEV), forced vital capacity (FVC), and FEV/FVC ratio, with values adjusted for age and sex in the two groups.

**Results:**

Among the 211 participants, 142 (67.3%) exhibited significant coronary artery involvement. Comparative analysis revealed no statistically significant differences in spirometric parameters [forced expiratory volume in one second (FEV_1_), FVC, FEV_1_/FVC ratio] between the groups. Notably, advancing age demonstrated a significant negative correlation with both FEF50% (β = -0.32, P < 0.01) and FVC values (β = -0.28, P < 0.05). Multivariate regression analysis identified inverse relationships between age and peak expiratory flow (PEF; β = -0.41, P < 0.001), HbA1C levels and PEF (β = -0.23, P < 0.05). Additionally, sex emerged as a significant modifier of %FEV_1_/FVC (β = 0.19, P < 0.05), with female participants showing lower ratios.

**Conclusions:**

This study failed to find an association between spirometry findings and coronary artery involvement. However, a significant relationship between small airway involvement and glycemic control was observed.

## 1. Background

The World Health Organization (WHO) reported a global diabetes prevalence of 8.5% among adults aged 18 years and older (422 million people) in 2014. By 2021, this number had risen by an additional 115 million cases (1). At present, the International Diabetes Federation (IDF) estimates that diabetes mellitus (DM) affects approximately 536.6 million adults worldwide, and that number is expected to increase to 783.2 million by 2045 (2). The DM confers a 5-fold increased risk of cardiovascular disease (CVD) (2, 3). Diabetes itself confers independent atherosclerotic cardiovascular disease (ASCVD) risk, and among people with diabetes, all major cardiovascular risk factors, including hypertension, hyperlipidemia, and obesity, are clustered and common (3, 4).

Chronic complications of uncontrolled hyperglycemia as a pathogenesis of diabetic complications are also highly complex. Hyperglycemia induces oxidative stress and excessive advanced glycation end (AGE) product formation. As the disease progresses, protein glycation and mitochondrial DNA (mtDNA) damage to respiratory chain components can, in turn, exacerbate oxidative stress injury. Elevated blood glucose stimulates inflammation, regulates immune cells, and promotes the production of cytotoxic free radicals, thereby attacking myocardial cells and vascular endothelial cells. This progressive pathophysiology results in multiorgan dysfunction (ocular, cardiovascular, pulmonary, neurological), significant disability, and premature mortality (4, 5).

While the exact mechanisms remain incompletely understood, uncontrolled hyperglycemia appears to drive systemic complications through chronic low-grade inflammation, oxidative stress from antioxidant depletion, and insulin resistance-mediated pathways (4-6). Insulin resistance in type 2 diabetes causes nonenzymatic protein glycation, accumulation of AGEs, activation of the AGE receptor (RAGE), and widespread organ damage (7).

The respiratory system exhibits distinctive pathology that includes structural changes, collagen/elastin deposition, and defects in connective tissue remodeling (8). In these patients, histopathological findings include basement membrane thickening, alveolar wall fibrosis, type II pneumocyte hyperplasia, and smooth muscle hypertrophy (8, 9). Considering that microvascular complications of diabetes, including lung involvement, are secondary to chronic inflammatory processes, it is important to note that, in addition to inflammatory processes, other factors such as lifestyle are involved in the occurrence of macrovascular complications.

## 2. Objectives

The present study was designed to investigate the relationship between these complications (5).

## 3. Methods

This study was a cross-sectional, one-center study conducted at Shahid Beheshti Hospital affiliated with Qom University of Medical Sciences from April 2019 to August 2021. We enrolled 211 diabetic patients who underwent coronary angiography (CAG) and met the inclusion criteria for the study. They were divided into two groups: Sixty-nine patients in group 1 [non-significant coronary artery disease (CAD)] and 142 patients in group 2 (significant CAD). Significant involvement of coronary arteries means involvement of main vessels (in the form of one main vessel, two main vessels, or three main vessels).

Inclusion criteria were age 30 - 75 years (inclusive), documented diagnosis of type 2 DM (based on ADA criteria) for more than 5 years before CAG, and absence of chronic pulmonary disease (confirmed by medical history and chest imaging). Exclusion criteria were smoking, previous cardiac surgery, known case of structural heart disease, anatomical deformity of the face, heart failure (EF less than 40%), uncontrolled hypertension, cardiac arrhythmia, history of lung diseases including recent pneumothorax, asthma, COPD, and bronchiectasis, and history of lung or thorax surgery. Eligible participants who met all inclusion and exclusion criteria were informed about the study details, and written informed consent was obtained from them.

Initially, a trained technician instructed participants on proper spirometry maneuvers. Spirometry was performed using a computerized spirometer, specifically the Spiroanalyzer ST-150 (FUKUDA SANGYO). The patient was seated comfortably, and the procedure was explained to them. Finally, three maneuvers meeting the repeatability and eligibility criteria (as per the ATS/ERS guidelines) were observed, and non-interpretable results were excluded per protocol. The spirometer-derived parameters were extracted for analysis. A normal pattern was defined as forced expiratory volume in one second (FEV_1_)/forced vital capacity (FVC) more than 70% and FVC more than 80%, with FEV_1_ more than 80% of predicted values; values below these thresholds indicated subnormal function.

Pulmonary function tests (PFT) help evaluate lung function, considering risk factors like occupational exposures. Results depend on patient effort and require clinical correlation. International guidelines have been established to standardize the interpretation of PFT results. Significant coronary stenosis was defined as more than 50% luminal narrowing in the left main coronary artery or more than 70% stenosis in major epicardial branches (LAD, LCx, RCA), as assessed by quantitative coronary angiography (QCA).

The statistical analysis included descriptive statistics, presented as mean ± SD or median [IQR] for continuous variables, and frequencies and percentages for categorical variables. Group comparisons were performed using independent *t*-tests, Mann-Whitney U tests, and chi-square or Fisher’s exact tests, as appropriate. Univariate and multivariate linear regression analyses were conducted for spirometric parameters, adjusting for age, sex, Body Mass Index (BMI), and diabetes duration, with assessments of multicollinearity (VIF less than 5), normality of residuals (Shapiro-Wilk test and Q-Q plots), and homogeneity of variance (Levene’s test). All analyses were performed using SPSS version 21 (IBM Corp). Two-tailed P-values less than 0.05 were considered statistically significant.

## 4. Results

A total of 211 patients were included in the study, with 69 patients in group I (non-significant CAD) and 142 patients in group II (significant CAD). There were no statistically significant differences in demographic characteristics between the two groups. The demographic variables (age, height, weight, BMI, HbA1C, and EF) for both groups are presented in Table 1. 

**Table 1.** Comparative Analysis of Baseline Characteristics and Pulmonary Function Tests ^a^

Variables	Group I ^b^; < 50% CAD	Group II ^c^; > 50% CAD	P-Value
**Total**	69	142	
**Sex**			0.877
Male	25	89	
Female	44	53	
**Age (y)**	58.71 ± 8.72	58.99 ± 9.37	0.838
**Height (cm)**	163.80 ± 11.24	159.40 ± 12.71	0.015
**Weight (kg)**	77.28 ± 12.51	75.58 ± 17.17	0.465
**BMI (kg/m** ^**2**^ **)**	28.98 ± 3.54	29.05 ± 5.97	0.926
**EF (%)**	49.86 ± 3.32	49.65 ± 3.13	0.659
**HbA1C (%)**	7.54 ± 0.75	7.48 ± 0.77	0.616
**MEF (L/s)**	85.83 ± 36.60	84.52 ± 41.59	0.825
**FVC (%)**	72.43 ± 17.13	73.45 ± 18.04	0.697
**FEV**_**1**_ ** (%)**	80.99 ± 22.65	81.24 ± 22.28	0.939
**FEV**_**1**_ **/FVC ratio**	89.07 ± 33.32	82.37 ± 26.88	0.118

Abbreviations: CAD, coronary artery disease; BMI, Body Mass Index; EF, ejection fraction; MEF, maximal expiratory flow; FVC, forced vital capacity; FEV_1_, forced expiratory volume in one second.

^a^ Values are expressed as mean ± SD.

^b^ Non-significant CAD.

^c^ Significant CAD.

The correlation between age, BMI, and hemoglobin A1C with spirometry parameters was assessed using the Pearson correlation test. There was a statistically significant correlation between age and PFEV_1_ (R = 0.181, P = 0.008), as well as HbA1C and maximal expiratory flow (MEF; R = -0.143, P = 0.037). The correlation of these three variables with other spirometry parameters was not statistically significant. The mean MEF in the group with more than 50% coronary artery involvement was 84.52 ± 41.59, while in the group with less than 50% involvement, it was 85.83 ± 36.60; this difference was not statistically significant (P = 0.825). Comparison of the mean values of other spirometry parameters, including PFVC, PFEV_1_, and PFEVFVC, also showed no significant differences between the two groups (Table 2). 

**Table 2.** Comparison of Spirometry Parameters Based on Coronary Artery Involvement ^a^

Variables	Coronary Artery Involvement		P-Value
	> 50%, Group I (69)	> 50%, Group II (142)	
**MEF**	85.83 ± 36.60	84.52 ± 41.59	0.825
**FVC**	72.43 ± 17.13	73.45 ± 18.04	0.697
**FEV**_**1**_	80.99 ± 22.65	81.24 ± 22.28	0.939
**FEV/FVC**	89.07 ± 33.32	82.37 ± 26.88	0.118

Abbreviations: MEF, maximal expiratory flow; FVC, forced vital capacity; FEV_1_, forced expiratory volume in one second; FEV/FVC, forced expiratory volume in one second divided by forced vital capacity.

^a^ Values are expressed as mean ± SD.

**Figure 1.**
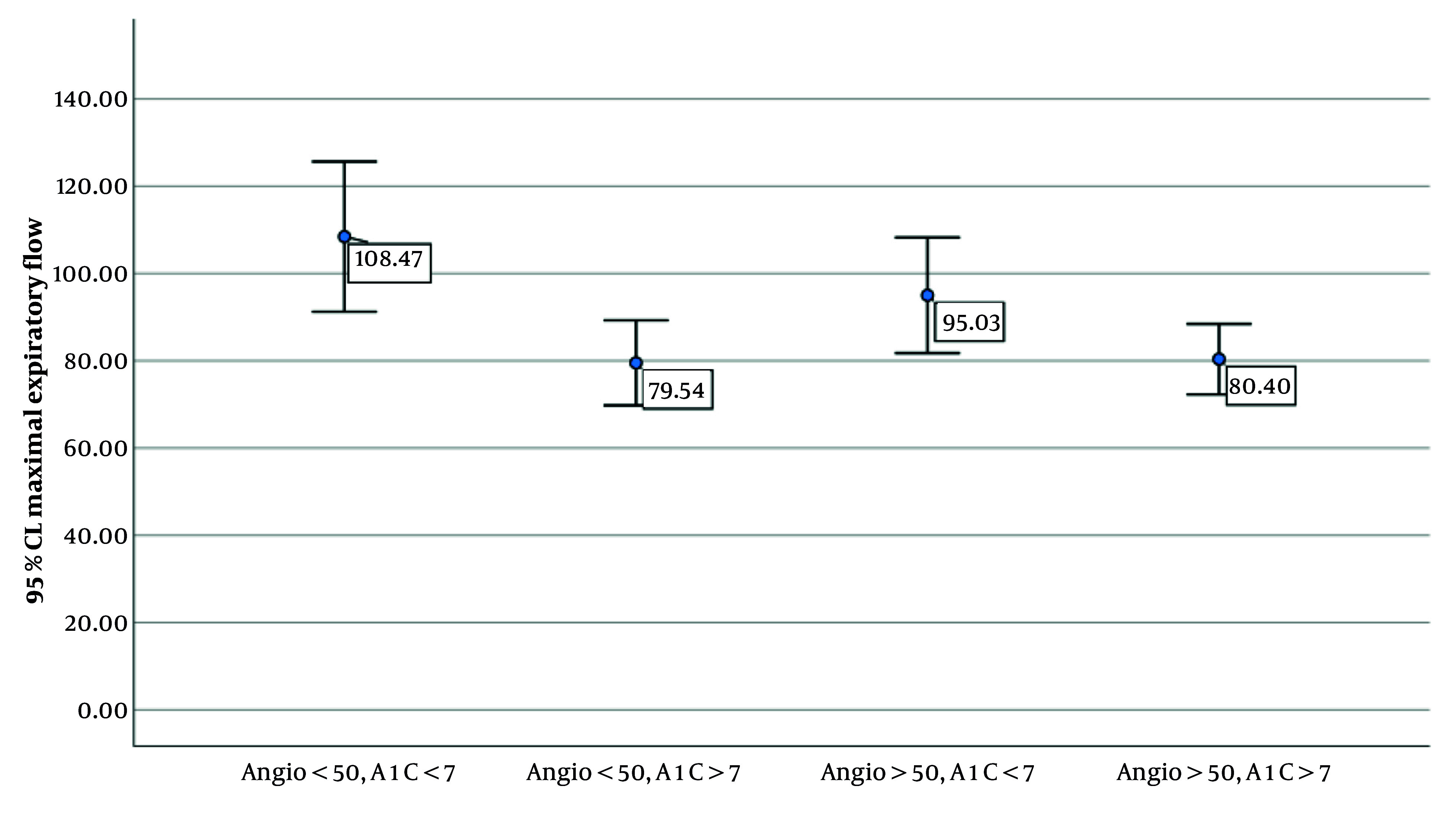
The comparison of maximal expiratory flow (MEF percent) among diabetic patients

Table 3 and Figure 1 present the comparison of MEF among diabetic patients categorized based on the severity of coronary artery involvement (< 50% vs. > 50%) and glycemic control status (A1C < 7 vs. A1C > 7). The results of the one-way ANOVA indicate a statistically significant difference in MEF across the groups (P = 0.017). Post-hoc pairwise comparisons revealed that patients with less than 50% coronary involvement and good glycemic control (A1C < 7) had significantly higher MEF values compared to those with poor glycemic control (A1C > 7), regardless of the degree of coronary involvement. Notably, MEF was highest in the group with both mild coronary involvement and well-controlled diabetes. These findings suggest a possible interaction between glycemic status and vascular involvement in determining pulmonary function in diabetic individuals.

**Table 3.** Comparison of Maximal Expiratory Flow Across Coronary Involvement and A1C Levels ^a^

**Coronary Artery Involvement**	**Hemoglobin A1C (%)**	**N**	**MEF (L/s); Mean **± **SD**	**Comparison Sub Group**	**P-Value ** ^**b**^
**Less than 50%**					
1	< 7	15	108 ± 31	2	0.12
2	> 7	54	80 ± 36	3	0.60
**More than 50%**					
3	< 7	40	95 ± 41	4	0.047
4	> 7	102	80 ± 41	1	0.010

Abbreviation: MEF, maximal expiratory flow.

^a^ Overall ANOVA = 0.17.

^b^ P-value between subgroups 2 and 3 = 0.60 and between subgroups 4 and 1 = 0.010.

Univariate regression showed that with each year increase in age, 0.038 units of MEF 50% (L/s) (P < 0.01) and 0.014 units of FVC (L/s, P < 0.001) are decreased. Multivariate regression of the relationship between %FEV/FVC and the investigated variables showed that only gender is related to %FEV/FVC, with this value being higher in women. Regression of the relationship between MEF and the investigated variables showed that only A1C is related to MEF, and this relationship is inverse; as A1C increases, the value of MEF decreases.

## 5. Discussion

The DM and its complications are a global health challenge. Early diagnosis of complications, especially with non-invasive methods, can help physicians manage diabetes. This study was designed based on the hypothesis that pulmonary function impairment in diabetic patients could serve as a non-invasive predictor of coronary artery involvement. The aim of this study was to evaluate the association between lung function and the level of glycemic control, as well as to investigate the relationship between microvascular (lung complications) and macrovascular (cardiovascular) complications in patients with type 2 diabetes. However, other variables, such as the relationship between diabetes control and pulmonary tests, were also examined.

In individuals with type 2 diabetes, factors such as microvascular impairment, chronic inflammatory processes, and glycosylation of lung tissues contribute to diminished pulmonary function, thereby increasing susceptibility to restrictive and obstructive lung disorders (10). On the other hand, although macrovascular mechanisms of diabetes are the main causes in the development of CAD (11), the development of macrovascular complications of diabetes is influenced not only by blood glucose levels but also by lifestyle factors (12).

Our study showed that pulmonary function test values did not differ significantly between patients with or without coronary artery involvement. However, regression analysis revealed a significant correlation between increased HbA1C levels and reduced MEF 50% (P = 0.41). Moreover, our study demonstrated that MEF 50% values differed significantly in both groups (with and without coronary artery involvement) when comparing patients with HbA1C levels greater than 7% to those with HbA1C levels less than 7%.

In our study, unlike the findings of other studies, no significant differences were found between PFT and cardiac complications (13-17). One of the reasons for the differences between the findings of our study and previous studies may be related to differences in sample size and study design. Moreover, the lack of a significant difference in PFT between patients with and without coronary artery involvement may be due to the presence of other factors that contribute to the development of macrovascular complications of diabetes but do not play a role in the occurrence of microvascular complications.

Our study demonstrated a significant difference in MEF values among all patients, regardless of coronary artery involvement, when comparing those with well-controlled blood glucose to those with poorly controlled levels. The reduction of MEF in patients with uncontrolled blood glucose may be consistent with the microvascular complications of diabetes. This finding aligns with the results of numerous previous studies (14, 18).

On the other hand, to the best of our knowledge, only a limited number of studies have failed to find an association between pulmonary function impairment and type 2 diabetes (2 g), which may be attributed to the small sample sizes in those studies. In conclusion, although pulmonary function impairments did not show a significant association with coronary artery involvement, the significant reduction in MEF among patients with uncontrolled diabetes compared to those with controlled diabetes indicates a potential relationship between small airway obstruction and diabetes. Finally, we recommend that studies with larger sample sizes and multicenter designs be conducted to confirm or refute the association between PFT and coronary artery involvement.

### 5.1. Conclusions

Although this study did not find an association between spirometry findings and coronary artery involvement, a significant relationship between small airway involvement and glycemic control was observed. Therefore, it is recommended that better diabetes control can prevent its pulmonary complications, and in patients with uncontrolled diabetes, it is also recommended that pulmonary function assessment be included in the clinical evaluation of these patients.

## Data Availability

The database displayed within the study is accessible upon request from the corresponding author during submission or after publication. The information is not freely accessible due to restrictions from the research institute.
